# Supplementary material for: Isolation and Molecular Characterisation of Respirovirus 3 in Wild Boar
Source: Animals (Basel). 2023 May 30;13(11):1815. doi: 10.3390/ani13111815 (PMC10252080; doi:10.3390/ani13111815)
Supplement: Supplementary file 1 [file animals-13-01815-s001.zip › animals-2407649-supplementary.pdf]

Supplemental Material for Publication.

1. Antigen detection enzyme-linked immunosorbent assay (ELISA)

1.1. MAbs production and selection

The BRV3 SF reference strain obtained from the American Type Culture Collection, Manassas, Va. (ATCC VR-281) was propagated in Madin-Darby bovine kidney (MDBK) cells grown in minimum essential medium Eagle (MEM). As soon as the cytopathic effect was fully developed, the virus was released from the cells by repeated freezing/thawing. After centrifugation (2000g for 15 min), the supernatant of the infectious culture medium was kept at -80 °C before being used as a positive control in the ELISA reaction. Negative control antigen was similarly prepared from non-infected cell lines. Monoclonal antibodies (MAbs) were prepared using standard methods (Galfre and Milstein, 1981). Mice were immunised with partially purified SF strain. Hybridomas were screened for secretion of desired antibodies by Indirect immunofluorescence (IFI) and indirect ELISA, set up using BRV3 infected and non-infected cells. MAbs specific for the BRV3 were selected and further characterised. Purified MAbs from hybridomas culture supernatants or ascitic fluids conjugated with horseradish peroxidase (HRPO) using a modification of the method described by Tjissen and Kurstak (1984). The different combinations of selected MAbs were tested in DAS-ELISA using as antigens both MDBK cells infected or not with BRV3 and cells infected with other paramyxoviruses or other bovine viruses. The intensity and specificity of the reaction using each MAb as catcher or tracer were considered as criteria for selection of the best combination, i.e., MAb 5F11 as antigen-catching antibody and 4A8 as conjugated MAb. In the standard procedure, Nunc Maxisorb Immunoplates were coated overnight at 4 °C with purified MAb 5F11 optimally diluted in 0.05 M carbonate–bicarbonate buffer, pH 9.6 (2 μg/well).

1.2. Antigen DAS ELISA

Samples were dispensed to duplicate wells and, after incubation a 37 °C for 1 h, the HRPO-conjugated MAb 4A8 was added at a pre-determined optimal dilution. Positive and negative controls for BRV3 were included in each plate. The diluting buffer consisted of PBS (pH 7.4) with 0.05% Tween 20 and 1% yeast extract. Following incubation at 37 °C for 1 h, the substrate solution (orth-ophenylenediamine dihydrochloride 0.5 mg/ml and 0.02% H2O2 in 50 mM phosphate citrate buffer, pH 5.0) was then added. The colorimetric reaction was stopped after 10 min by the addition of 2 N sulphuric acid, and the absorbance values were read at 492 nm using a spectrophotometer. Results were expressed as an optical density (OD). Fifty microliters per well of reagent were dispensed; three washing steps with PBS-Tween 20 were performed after each incubation. The dilutions 1/1 and 1/2 provided the widest window between them and were selected as screening dilutions combined with an OD cut-off value of 0.2.

2. Hemagglutination test

Hemagglutination tests were performed with 0.25% guinea pig erythrocyte suspension in 96-well V-bottom plates. Equal volumes (0.5 ml) of serial twofold dilutions of the virus isolates for hemagglutinin activity and erythrocyte suspension were shaken and allowed to sediment for 45 min at 4 °C. The diluent in all tests was 0.01 M PBS at a pH of 7.4. Read the endpoint by tilting the plate at 45° for 30 s. The endpoint is the last well in which the guinea pig erythrocytes do not run, and the titers were recorded (Zhu et al., 2011; WOAH, 2021).

References

Galfre, G., Milstein, C., 1981. Preparation of monoclonal antibodies, strategies and procedure. Methods in Enzymology 75, 3–53

Tjissen, P., Kurstak, E., 1984. Highly efficient and simple methods for the preparation of peroxidase and active peroxidase–antibodies conjugates for enzyme immunoassays. Analytical Biochemistry 136, 451–457.

Zhu YM, Shi HF, Gao YR, Xin JQ, Liu NH, Xiang WH, Ren XG, Feng JK, Zhao LP, Xue F. Isolation and genetic characterization of bovine parainfluenza virus type 3 from cattle in China. Vet Microbiol. 2011 May 5;149(3-4):446-51. doi: 10.1016/j.vetmic.2010.11.011.

WOAH Manual for Terrestrial Animals 2021 cap 3.3.14 par B1.3: Newcastle disease (infection with Newcastle disease virus) – Diagnostic techniques – Detection of the agent – Virus identification. Available online: https://www.woah.org/fileadmin/Home/eng/Health_standards/tahm/3.03.14_NEWCASTLE_DIS.pdf
